# Prescription Trends and Clinical Decision‐Making in Neuropathic Pain Pharmacological Treatment: Results From a Cross‐Sectional Survey by the Spanish Pain Society

**DOI:** 10.1002/ejp.70246

**Published:** 2026-03-10

**Authors:** Miguel Á. Huerta, Mónica Mayo‐Moldes, Miguel M. Garcia, Beliu García‐Parra, Mercè Matute, Yolanda López‐Tofiño, Nancy Paniagua, Mar Hernández‐Secorún, Dolors Soler, Marcos Salmerón, Julian Taylor, Enrique Verdú, José A. Valencia, Silvia Pico, Clara Díaz‐Alejo, Masahito Katsuki, Ancor Serrano‐Afonso, M. Carmen Ruiz‐Cantero

**Affiliations:** ^1^ Neuropathic Pain Working Group Spanish Society of Pain (GTDN‐SED) Madrid Spain; ^2^ Department of Pharmacology University of Granada Granada Spain; ^3^ Biosanitary Research Institute ibs.GRANADA Beiro (Granada) Spain; ^4^ Pain Unit, Service of Anaesthesiology and Resuscitation, University Hospital Complex of Vigo Galician Healthcare Service (SERGAS) Vigo Pontevedra Spain; ^5^ Area of Pharmacology, Nutrition and Bromatology, Department of Basic Health Sciences, Unidad Asociada de I+D+i al Instituto de Química Médica (IQM) CSIC‐URJC Universidad Rey Juan Carlos Alcorcón (Madrid) Spain; ^6^ High Performance Experimental Pharmacology Research Group Universidad Rey Juan Carlos (PHARMAKOM) Alcorcón (Madrid) Spain; ^7^ High Performance Environmental Risk for Health and the Environment Research Group Universidad Rey Juan Carlos (RIsAMa) Alcorcón (Madrid) Spain; ^8^ Clinical Neurophysiology Section, Neurology Service, Hospital Universitari de Bellvitge Universitat de Barcelona‐Health Campus, IDIBELL L'Hospitalet de Llobregat (Barcelona) Spain; ^9^ Pain Unit, Hospital Universitari Dexeus‐Grupo Quirónsalud Barcelona Spain; ^10^ High Performance Research Group in Physiopathology and Pharmacology of the Digestive System Universidad Rey Juan Carlos (NEUGUT) Alcorcón (Madrid) Spain; ^11^ Physiotherapy Research Unit, Faculty of Health Science Universidad de Zaragoza Zaragoza Spain; ^12^ Spinal Cord Injury, Neurorehabilitació Guttmann Hospital Universidad Autònoma de Barcelona Badalona Barcelona Spain; ^13^ Chronic Pain Unit Hospital Universitario Virgen de las Nieves Beiro (Granada) Spain; ^14^ Sensorimotor Function Group Hospital Nacional de Parapléjicos (SESCAM) Toledo Spain; ^15^ Instituto de Investigación Sanitaria de Castilla‐La Mancha (IDISCAM) Toledo Spain; ^16^ Harris Manchester College University of Oxford Oxford UK; ^17^ Research Group of Clinical Anatomy, Embryology and Neuroscience (NEOMA), Department of Medical Sciences Universitat de Girona Girona Spain; ^18^ Service of Anaesthesia, Reanimation and Pain Treatment Hospital General Universitario Reina Sofía de Murcia Murcia Spain; ^19^ Pain Unit Hospital Universitario Rio Hortega Valladolid Spain; ^20^ Service of Anaesthesia, Reanimation and Pain Treatment Hospital Clínico San Juan de Alicante Sant Joan d'Alacant (Alicante) Spain; ^21^ Physical Education and Health Center Nagaoka University of Technology Niigata Japan; ^22^ Insight Research Ireland Centre for Data Analytics and School of Health and Human Performance Dublin City University Dublin Ireland; ^23^ Department of Anaesthesia, Reanimation and Pain Clinic, Hospital Universitari de Bellvitge Universitat de Barcelona‐Health Campus, IDIBELL L'Hospitalet de Llobregat (Barcelona) Spain; ^24^ Department of Pharmacology, Toxicology and Therapeutic Chemistry Universitat de Barcelona Barcelona Spain

**Keywords:** clinical practice, drug prescriptions, guideline, health care surveys, neuralgia, tolerance

## Abstract

**Background:**

Neuropathic pain (NP) is a complex chronic condition with limited therapeutic effectiveness. Despite multiple drug classes and international guidelines, real‐world adherence remains inconsistent, and data on prescribing practices among pain specialists is scarce.

**Methods:**

We conducted a nationwide cross‐sectional survey among members of the Spanish Pain Society in May 2025. A structured 62‐item questionnaire assessed prescribing habits, decision‐making criteria, guideline adherence, dosage patterns, and the recognition and management of tolerance. Sociodemographic and professional data were also collected.

**Results:**

A total of 220 pain specialists (52% female) completed the survey; 28% had over 20 years of clinical experience. Satisfaction with current pharmacological options was modest, with 52% reporting dissatisfaction or indifference. Prescribing was mainly guided by clinical experience (43%) and guideline recommendations (36%). Gabapentin (45%) and pregabalin (40%) were the most frequent first‐line choices, followed by tricyclic antidepressants (amitriptyline; 9%), duloxetine (5%) and venlafaxine (1%). Tramadol dominated second‐line use (65%), followed by capsaicin (22%) or lidocaine (5%) patches. Half of the participants reported tolerance, typically after 3–12 months, managed mainly by dose escalation or switching drug classes. Dosage practices for gabapentinoids and antidepressants showed marked heterogeneity, with frequent deviations from recommended titration protocols.

**Conclusions:**

NP management in Spain shows variability and partial alignment with international guidelines. Gabapentinoids, tricyclic antidepressants and duloxetine remain preferred treatments, but reliance on personal experience and awareness of tolerance hinder evidence‐based practice. Quantifying Spanish pain specialists' views on tolerance supports calls for national consensus, better dosing education and further research to harmonise care and improve outcomes.

**Significance:**

This nationwide survey provides the first systematic assessment of neuropathic pain management in Spain, revealing marked variability, only partial adherence to international guidelines, and persistent reliance on clinical experience over evidence. It confirms gabapentinoids, tricyclic antidepressants and duloxetine as preferred treatments, identifies continued tramadol use despite conflicting recommendations, and quantifies for the first time clinicians' perception and management of tolerance. These findings fill a major evidence gap and directly inform future national consensus, educational initiatives and clinical practice improvement.

## 1 Introduction

Neuropathic pain (NP) refers to chronic pain caused by a lesion or disease of the somatosensory nervous system (Jensen et al. 2011), in accordance with the classification provided in ICD‐11 (Scholz et al. 2019). It is among the most complex chronic pain conditions, with an estimated prevalence of 7%–10% in the general population (Van Hecke et al. 2014), and even higher rates in specific patient groups, including those with diabetes (Gylfadottir et al. 2020), multiple sclerosis (Rodrigues et al. 2023), herpes zoster (Huerta et al. 2023) or cancer (Roberto et al. 2016). Beyond epidemiology, NP imposes profound functional, psychological and social consequences (Inoue et al. 2017) and represents a considerable economic burden through increased healthcare utilisation, polypharmacy and productivity loss (McDermott et al. 2006; Doth et al. 2010; Andrew et al. 2014; Liedgens et al. 2016).

Pharmacological treatment remains a major challenge. Despite the availability of several drug classes—including gabapentinoids, serotonin–noradrenaline reuptake inhibitors (SNRIs), tricyclic antidepressants (TCAs), topical agents and selected opioids, the analgesic effects are often modest. Evidence from randomised controlled trials and meta‐analyses indicates that fewer than 30%–40% of patients achieve clinically meaningful pain relief with monotherapy (Finnerup et al. 2015; Soliman et al. 2025). Furthermore, treatment tolerability, comorbidities and drug accessibility complicate long‐term management, frequently requiring therapy modifications.

International guidelines, such as those issued by the Neuropathic Pain Special Interest Group (NeuPSIG) (Finnerup et al. 2015; Soliman et al. 2025), the National Institute for Health and Care Excellence (NICE 2020) and the European Federation of Neurological Societies (EFNS; Attal et al. 2010), provide structured, evidence‐based recommendations. However, adherence in real‐world practice frequently remains inconsistent. Contributing factors include limited perceived efficacy, heterogeneity of populations, local prescribing restrictions and a strong reliance on clinical experience. The restrictive inclusion criteria of randomised trials, often excluding patients with multimorbidity and polypharmacy, further exacerbates the evidence‐practice gap (Bouhassira and Attal 2016; Soliman et al. 2025). Clinical uncertainty is also amplified by conflicting guidance, such as the endorsement of weak opioids (e.g., tramadol) in some guidelines (EFNS, NeuPSIG; Sommer et al. 2020) versus their discouragement by the NICE. Moreover, international surveys reveal substantial discrepancies between guidelines and real‐world prescription patterns (Hans et al. 2007; Gustavsson et al. 2013; Martinez et al. 2014; Callaghan et al. 2019; Machado‐Duque et al. 2020; Singh et al. 2020; Serrano Afonso et al. 2021; Rault et al. 2025). Yet, despite these global insights, systematic evidence regarding analgesic prescription practices in Spain remains scarce, underscoring the need for country‐specific data.

A particularly underexplored issue is tolerance, the weakening of drug efficacy over time (Svensson 2022). Although frequently observed in routine practice, tolerance to analgesic treatment has rarely been evaluated in randomised trials. Better characterisation of this phenomenon could provide important insights into treatment dissatisfaction and frequent therapy adjustments. Taken together, these gaps highlight the importance of understanding prescribing habits, the role of guidelines, and decision‐making criteria of Spanish specialists.

Therefore, the aim of this study was to investigate prescribing trends, decision‐making strategies, and approaches to managing NP—including tolerance—among members of the Spanish Pain Society, and to assess their alignment with international guidelines.

## 2 Methods

### 2.1 Study Design

This study was designed as a descriptive, cross‐sectional survey. An electronic questionnaire was used to capture real‐world prescribing habits and clinical decision‐making criteria in the management of NP among Spanish pain specialists (see below). The primary objective was to provide a comprehensive description of analgesic prescribing patterns, decision‐making strategies and management approaches, with a particular emphasis on their alignment with international guidelines. In addition, the study aimed to quantify, for the first time, the clinicians' perception and management of tolerance.

### 2.2 Participants

Eligible participants were physicians affiliated with the Spanish Pain Society (SED) or professionals working in pain units across Spain, regardless of their medical specialty. Inclusion criteria for pain experts were: (1) active clinical practice with direct involvement in the management of patients with NP, and (2) willingness to voluntarily complete the survey. Responses with more than 30% missing data were excluded from the final analysis. Participation was anonymous, and no personal identifiers were collected.

### 2.3 Survey Instrument

A structured 62‐item questionnaire was designed by the Neuropathic Pain Working Group of the SED (full version available in Supporting Information). The instrument was developed through an iterative expert consensus process and piloted by five clinicians to ensure clarity and content validity (M.M.‐M., B.G.‐P., M.M., D.S. and A.S.‐A.). The questionnaire was comprised of four thematic sections: (1) General prescribing habits and satisfaction with current pharmacological treatments; (2) Specific prescribing patterns by therapeutic line, classified according to international recommendations NeuPSIG (Dworkin et al. 2007): first‐line (gabapentinoids, SNRIs, TCAs), second‐line (tramadol, lidocaine 5% patch, capsaicin 8% patch), third‐line (botulinum toxin A, opioids), and subsequent options; (3) Dosing and adjustment practices for commonly used drugs (gabapentin, pregabalin, duloxetine, venlafaxine, TCAs), including starting dose, titration scheme, maximum dose, and use of extended‐release formulations and (4) Sociodemographic and professional characteristics (age, gender, years of prescribing experience, specialty, geographic region and practice setting), which may influence prescribing behaviour.

The survey included multiple‐choice, single‐choice and ranking questions, as well as limited open‐ended items to capture the rationale underlying therapeutic choices (e.g., preference for a particular antiepileptic or antidepressant).

### 2.4 Data Collection

The survey was conducted in Spain in May 2025. Recruitment and data collection took place during the annual congress of the Spanish Pain Society (SED) held in Málaga (28–30 May 2025). In parallel, the questionnaire was distributed through the Society's official communication channels, including newsletters and targeted mailing lists, to maximise participation across regions. All responses were collected electronically using a secure online platform compliant with national and European data protection regulations. The survey software was configured so that only one response per IP address was allowed, minimising the likelihood of duplicate participation. Participation was voluntary and anonymous, and no personal identifiers were recorded.

### 2.5 Statistical Analysis

Data were analysed using GraphPad Prism software (v8.0; GraphPad Software Inc., USA). Categorical variables were summarised as absolute frequencies (*n*) and percentages (%). For binary outcomes, proportions were compared against a theoretical 50% distribution using a binomial test. For categorical outcomes with more than two levels, goodness‐of‐fit Chi‐squared tests were used to assess deviations from a uniform distribution. Associations between clinicians' professional characteristics and prescribing behaviours were explored using Pearson's Chi‐squared tests. Years of prescribing experience and frequency of NP management (expertise in NP management) were considered predictor variables. Outcomes included adherence to clinical guidelines, choice of first‐line pharmacological agent, and preference for tramadol as a second‐ or third‐line treatment. When expected cell counts were low, *p*‐values were estimated using Monte Carlo simulation (5000 permutations). Effect sizes were estimated using Cramér's *V*.

### 2.6 Ethical Considerations

The study adhered to the principles of the Declaration of Helsinki. Participation was anonymous, voluntary and non‐remunerated. Given the absence of patient‐level data and the non‐interventional design, formal approval from a research ethics committee was deemed unnecessary under Spanish and European regulations (Regulation EU 2016/679, GDPR; Organic Law 3/2018 on Personal Data Protection).

## 3 Results

### 3.1 Sociodemographic Characteristics of Survey Participants

A total of 220 physicians completed the survey and were included in the final analysis. The survey was disseminated to all members of the SED and clinicians working in pain units across Spain; however, the exact number of potentially eligible physicians could not be determined. Eligibility required active clinical involvement in the management of patients with NP. No follow‐up was conducted due to the cross‐sectional design.

The sociodemographic characteristics of the 220 respondents are summarised in Table 1. Slightly more than half were female (52.3%), 42.7% were male, and a minority identified as other (0.9%) or preferred not to disclose gender (4.1%). Regarding age, 13.9% were under 30 years, 24.1% were 31–40 years, 23.6% were 41–50 years, 22.7% were 51–60 years, 13.9% were 61–70 years, and 1.8% were older than 70 years. In terms of geographic distribution, most respondents were based in Catalonia (14.5%), the Community of Madrid (12.3%), Andalusia (10.5%), and the Valencian Community (6.8%). Foreign responses accounted for 8.6%, corresponding to international locations in Europe and Latin America, where experts maintain close professional ties with the Spanish Pain Society. Additionally, 26.8% of respondents did not specify their location. Regarding professional experience, 27.8% had more than 20 years in practice, 24.5% reported 11–20 years, 13.4% had 6–10 years, and 34.3% had ≤ 5 years.

**TABLE 1:** Sociodemographic characteristics of survey participants.

Variable	Category	Frequency	Percentage
Gender	Female	115	52.3
	Male	94	42.7
	Prefer not to say	9	4.1
	Other	2	0.9
Age	≤ 30 years	30	13.9
	31–40 years	52	24.1
	41–50 years	51	23.6
	51–60 years	49	22.7
	61–70 years	30	13.9
	> 70 years	4	1.8
Geographic location	Catalonia	32	14.5
	Community of Madrid	27	12.3
	Andalusia	23	10.5
	Valencian Community	15	6.8
	Galicia	11	5.0
	Region of Murcia	9	4.1
	Castilla–La Mancha	4	1.8
	Basque Country	4	1.8
	Aragon	2	0.9
	Castilla y León	2	0.9
	Extremadura	1	0.5
	Balearic Islands	1	0.5
	Navarre	2	0.9
	Canary Islands	8	3.6
	Ceuta	1	0.5
	Foreign	19	8.6
	Unspecified	59	26.8
Clinical experience	≤ 5 years	74	34.3
	6–10 years	29	13.4
	11–20 years	53	24.5
	> 20 years	60	27.8

### 3.2 Prescribing Patterns and Factors for Clinical Decision‐Making

**FIGURE 1:**
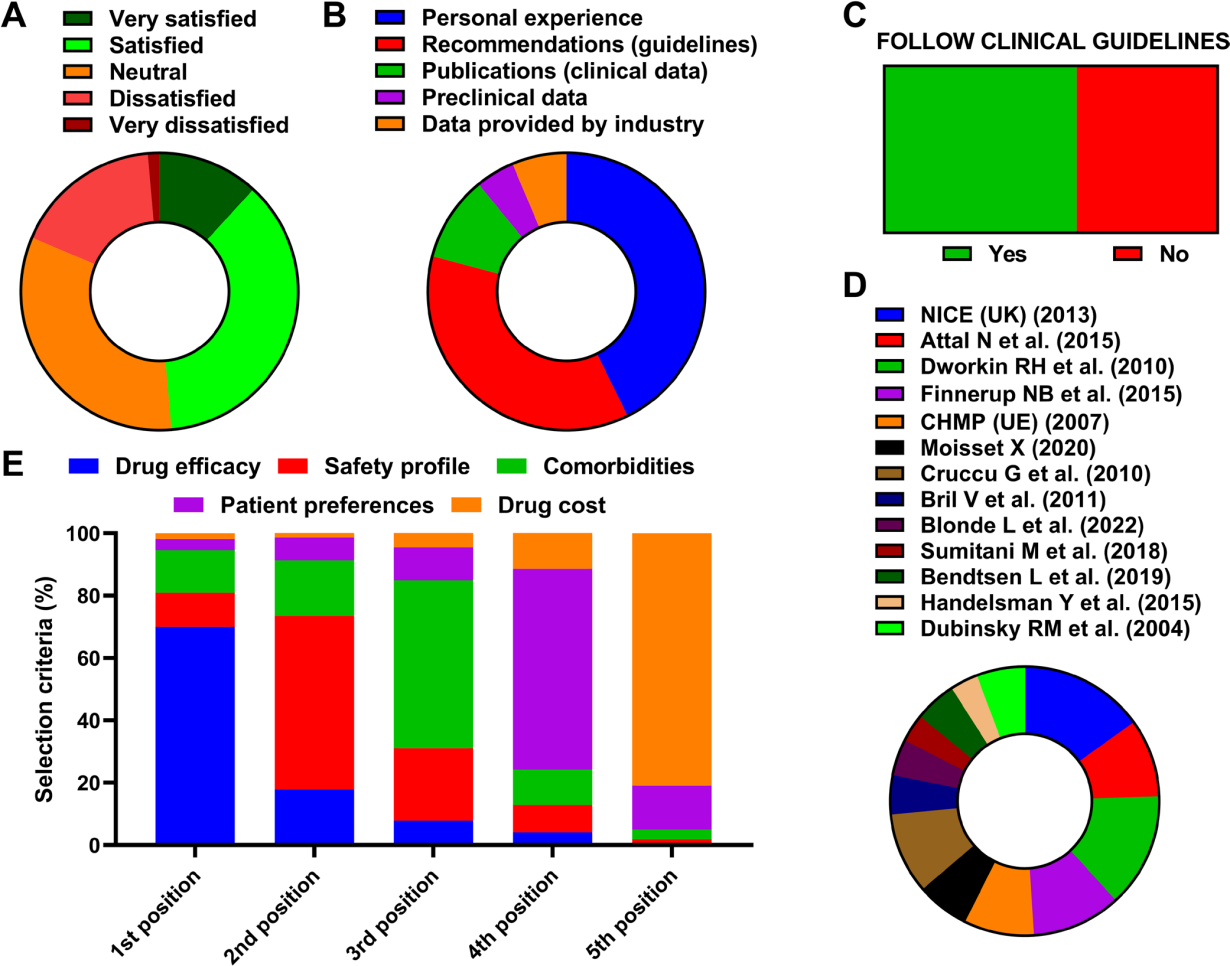
Prescription patterns. (A) Overall satisfaction with pharmacological treatments for neuropathic pain (*n* = 220). (B) Main factors for prescribing decisions, including personal experience, guideline recommendations, publications, preclinical data and information provided by industry (*n* = 220). (C) Proportion of respondents reporting adherence to clinical guidelines in daily practice (*n* = 220). (D) Specific guidelines most frequently cited, including NICE (2013; version indicated in the survey; last updated 2020), NeuPSIG (Dworkin et al. 2007) and Dworkin et al. (2010) (*n* = 298 responses, multiple‐choice question). (E) Ranked criteria influencing drug selection, including efficacy, safety profile, comorbidities, patient preferences and drug cost.

Among the 220 respondents, overall satisfaction with current pharmacological options for NP was modest (Figure 1A). Only 11.8% reported being very satisfied, while 36.8% were satisfied, 32.7% expressed a neutral opinion, and nearly half reported dissatisfaction (17.3% dissatisfied, 1.4% very dissatisfied). The main factors for prescribing decisions were personal clinical experience (42.7%) and clinical guideline recommendations (36.4%), compared to scientific publications (10.0%), preclinical evidence (4.5%), and industry‐provided data (6.4%) that were cited infrequently (Figure 1B). Regarding guideline adherence, most participants (57.7%; *p* = 0.020) reported following clinical practice guidelines in their daily practice, while 42.3% did not (Figure 1C). Among those adhering to guidelines, the most frequently cited were NICE (NICE 2013; version indicated in the survey; last updated 2020; 15.1%), Dworkin et al. (2010; 13.8%), and NeuPSIG (Finnerup et al. 2015; 10.7%) (Figure 1D). Other guidelines, including EFNS, French recommendations, and American Academy of Neurology statements, were mentioned less frequently. When ranking factors influencing drug selection, therapeutic efficacy was overwhelmingly the most important first‐choice criterion (70.0%). This was followed by safety profile (59.1% ranked second), comorbidities (56.8% ranked third), patient preferences (63.2% ranked fourth), and drug cost, which was most often considered only in last position (80.9%) (Figure 1E).

### 3.3 Treatment Modification and Management of Analgesic Tolerance

**FIGURE 2:**
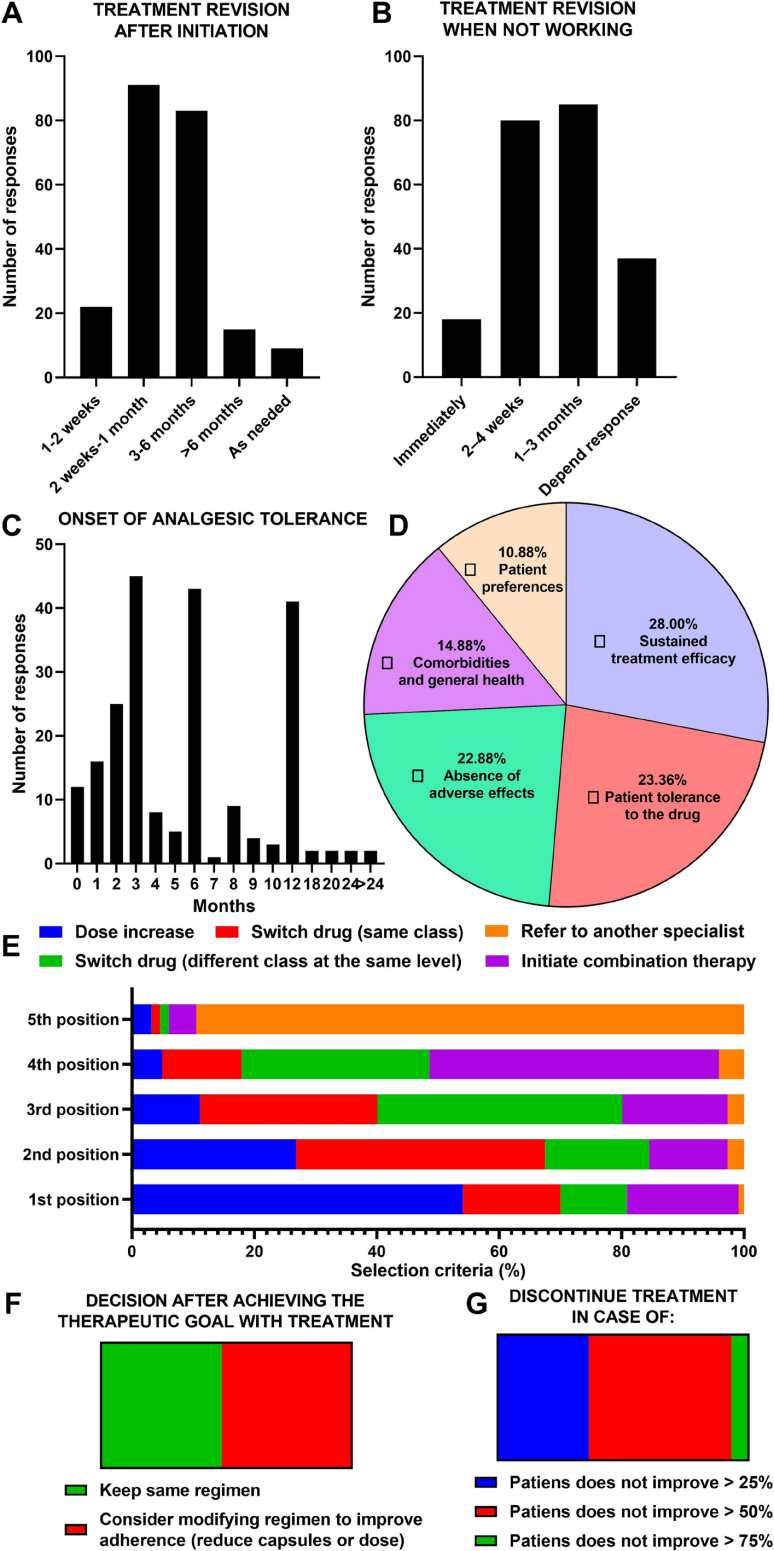
Treatment modification patterns. (A) Timing of treatment reassessment after initiation (*n* = 220). (B) Timing of reassessment when treatment is not working (*n* = 220). (C) Reported onset of tolerance, expressed in months since treatment initiation (*n* = 220). (D) Ranked criteria for treatment continuation, including sustained efficacy, tolerance, absence of adverse effects, comorbidities, and patient preferences. (E) Preferred strategies to manage tolerance, including dose escalation, switching within or across drug classes, initiating combination therapy, or referral (*n* = 220). (F) Decision after achieving therapeutic goals: Maintenance of the same treatment regimen versus modifications to improve adherence (*n* = 220). (G) Thresholds for treatment discontinuation according to percentage of patient improvement (*n* = 220).

**FIGURE 3:**
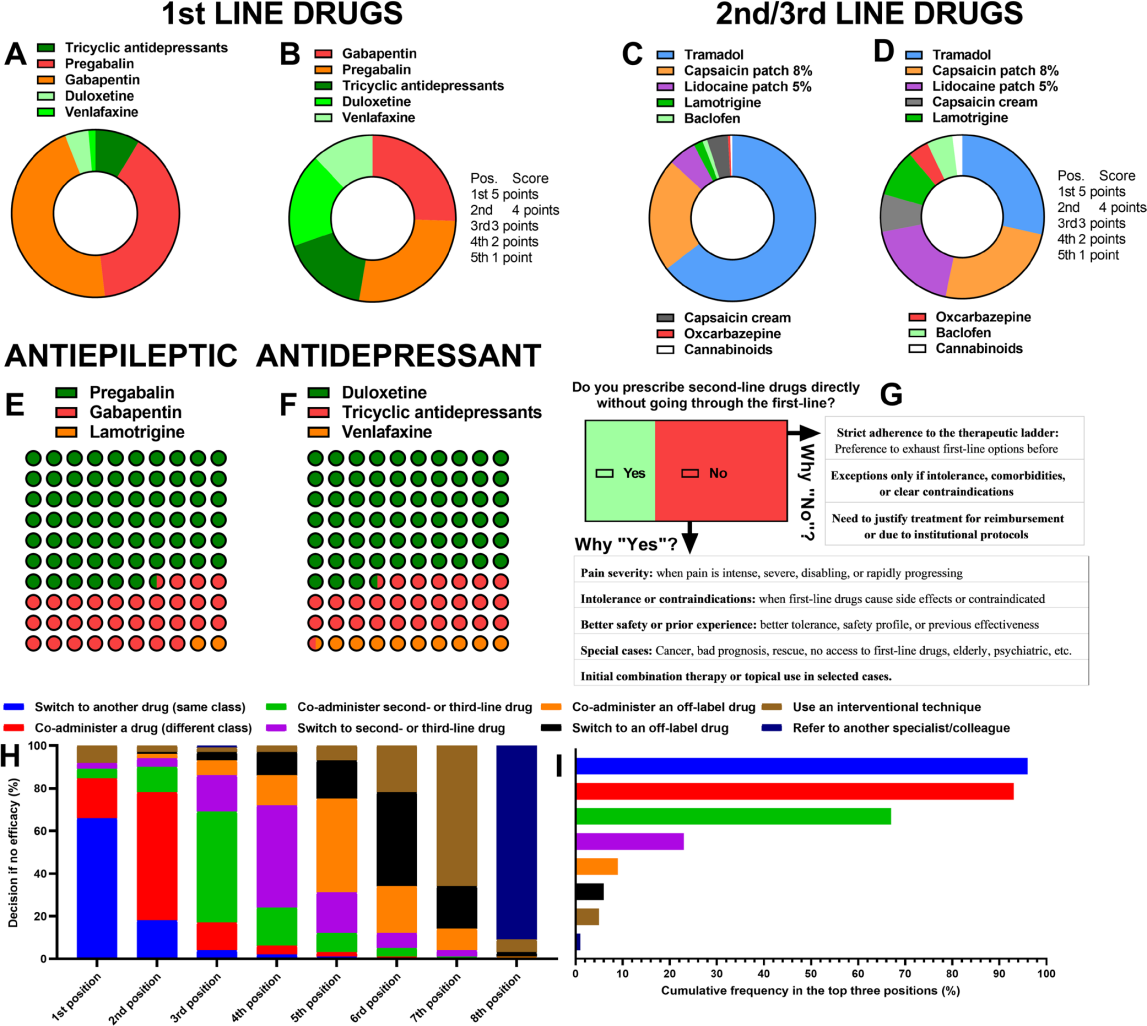
Preferences for first‐, second‐, and third‐line drug classes. (A, B) First‐line treatments ranked by frequency and weighted score (gabapentin, pregabalin, tricyclic antidepressants, duloxetine, venlafaxine). (C, D) Preferred antiepileptic and antidepressant classes for neuropathic pain. (E, F) Second‐ and third‐line agents (tramadol, capsaicin 8% patch, lidocaine 5% patch, lamotrigine, oxcarbazepine, baclofen, cannabinoids). (G) Proportion of specialists prescribing second‐line drugs without prior use of first‐line options and rationale for this choice. (H, I) Preferred strategies in cases of insufficient treatment effectiveness, including within‐class analgesic switches, class substitution, addition of second‐ or third‐line drugs, or off‐label combinations.

Most respondents reported reassessing treatment response between 2 weeks and 6 months after initiation, whereas only a minority adjusted therapy either very early (1–2 weeks) or waited beyond 6 months (Figure 2A). When treatment was deemed ineffective, most respondents reported modifying therapy within 2 weeks to 3 months, while only a minority adjusted it immediately or tailored changes solely to the patient's individual response (Figure 2B). Tolerance was reported by half of respondents (50.0%), most frequently emerging between 3 and 12 months after treatment initiation (Figure 2C). When ranking criteria for treatment continuation or discontinuation, sustained efficacy (28.0%) was consistently identified as the most decisive factor, followed by patient tolerance (23.4%) and absence of adverse events (22.9%) (Figure 2D). Comorbidities or overall health status (14.9%), and patient preferences (10.9%) were considered important but ranked lower. Management strategies for tolerance varied considerably (Figure 2E). The most frequent approach was dose escalation, followed by switching to another drug within the same class, or to a different class at the same therapeutic level. Combination therapy and referral to another specialist were less commonly prioritised (Figure 3E). After achieving the therapeutic goal, respondents were evenly split (*p* = 0.592) between maintaining the established treatment regimen (48.2%) and modifying it to improve adherence (e.g., reducing capsule burden or using extended‐release formulations; 51.8%) (Figure 2F). Clinicians showed a clear preference regarding the threshold for discontinuing treatment in case of insufficient response (Figure 2G). Most respondents reported discontinuing therapy when patients failed to achieve at least a 50% improvement (56.8%), whereas fewer selected thresholds of > 25% (36.4%) or > 75% (6.8%). This distribution differed significantly from a uniform distribution (*χ* ^2^(2) = 70.9, *p* < 0.001), with a large effect size (Cramér's *V* = 0.57).

### 3.4 Preferences for First‐, Second‐ and Third‐Line Drug Classes

When examining first‐line therapies, gabapentin and pregabalin clearly emerged as the predominant options (*p* < 0.001; Cramér's *V* = 0.47) (Figure 3A). Respondents showed a similar preference for gabapentin and pregabalin (*p* = 0.306) when considering first‐choice selection (45.9% and 39.5%, respectively), a pattern that remained consistent in the weighted scoring analysis (25.5% and 27.1%, respectively) (Figure 3B). Antidepressants were selected far less frequently as first‐choice options (TCAs: 8.6%; duloxetine: 4.5%; venlafaxine: 1.4%), although their relative contribution increased notably in the weighted analysis (TCAs: 17.0%; duloxetine: 18.4%; venlafaxine: 12.0%).

For second‐ and third‐line treatments, tramadol was the most frequently selected option (*p* < 0.001; Cramér's *V* = 0.48), chosen as the first preference by 64.5% of respondents and achieving the highest cumulative weighted score (28.5%) (Figure 3C,D). The capsaicin 8% patch followed, with 22.3% first‐choice selection and 24.8% of the weighted score, typically ranking within the top two positions. Lidocaine patch reached 5.5% first‐choice selection and 18.7% in the weighted analysis, while lamotrigine (1.8% first‐choice selection) and capsaicin cream (4.1% first‐choice selection) accounted for 9.4% and 7.5% of the weighted score, respectively. Other treatments such as oxcarbazepine, baclofen, and cannabinoids were rarely prioritised and predominantly appeared in the lowest ranks.

Concerning class‐specific preferences, pregabalin was the preferred antiepileptic agent for NP (*p* < 0.001), reported by 66.4% of respondents, while gabapentin was preferred by 31.8% and lamotrigine by 1.8% (Figure 3E). For antidepressants (Figure 3F), duloxetine was the leading choice (63.6%; *p* < 0.001), followed by TCAs (amitriptyline; 26.8%) and venlafaxine (9.5%).

Most specialists (65.5%; *p* < 0.001) reported adhering to a stepwise therapeutic approach, exhausting first‐line options before prescribing second‐line drugs (Figure 3G). Nonetheless, a relevant proportion (34.5%) acknowledged initiating second‐line agents directly in selected scenarios, such as severe or rapidly progressing pain, intolerance or contraindications to first‐line drugs, previous evidence of effectiveness in the same patient, or special clinical contexts including cancer, frailty, or psychiatric comorbidities.

When first‐line therapy failed, the most common strategy was to switch to another drug within the same pharmacological class, followed by switching to a different class or introducing a second‐ or third‐line agent (Figure 3H). Combination therapy, off‐label drug use, and referrals to other specialists were less frequently reported. Cumulative ranking of preferred strategies confirmed within‐class switching and class substitution as the dominant approaches, followed by co‐administration of additional agents (Figure 3I).

### 3.5 Antiepileptic Drug Dosing Patterns

**FIGURE 4:**
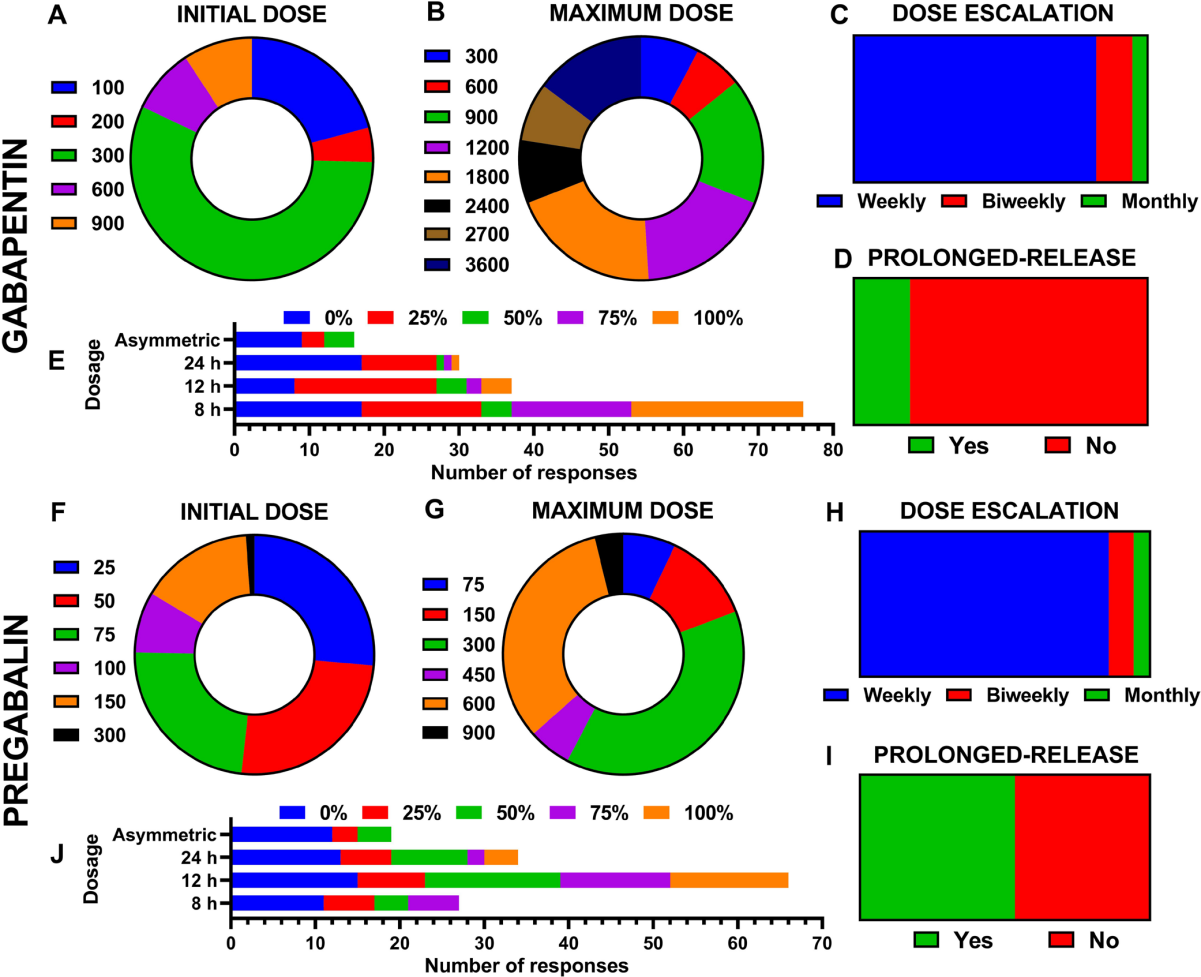
Dosage patterns of antiepileptic drugs. (A–E) Gabapentin: Initial and maximum doses (mg/day), titration schedules, use of prolonged‐release drug formulations, and preferred dosing frequency. (F–J) Pregabalin: Initial and maximum doses (mg/day), titration schedules, use of prolonged‐release formulations, and preferred dosing frequency.

Most clinicians reported initiating gabapentin at 300 mg (56.6%), whereas lower (100–200 mg; 20.8% and 4.6%, respectively) and higher doses (≥ 600 mg; 8.7%) were selected less frequently. This distribution differed significantly from a uniform distribution (*χ* ^2^(4) = 166.8, *p* < 0.001), with a large effect size (Cramér's *V* = 0.69). In contrast to the clearly standardised pattern observed for initial dosing, reported maximum gabapentin doses showed a broader distribution across categories (Figure 4B). Although clinicians most frequently reached maximum doses between 1200 and 1800 mg (18.1% and 20.0%, respectively), substantial variability was observed, with both lower (900 mg; 16.8%) and higher (2400, 2700 or 3600 mg; 8.4%, 7.7% and 14.8%, respectively) dose ceilings being selected. Overall, the distribution of maximum doses differed significantly from a uniform pattern (*χ* ^2^(7) = 26.0, *p* < 0.001), albeit with a small‐to‐moderate effect size (Cramér's *V* = 0.16), reflecting heterogeneity in dose escalation strategies once treatment was established. Dose escalation was generally performed on a weekly basis (*p* < 0.001; Cramér's *V* = 0.73), while biweekly or monthly adjustments were seldom applied (Figure 4C). Most respondents reported not using prolonged‐release gabapentin formulations (*p* < 0.001), noting that these are not commercially available in Spain and therefore standard immediate‐release regimens are routinely employed (Figure 4D). Gabapentin dosing frequency showed limited variability, with administration every 8 h (three‐times‐daily) being clearly the most common regimen, whereas 12‐h and once‐daily schedules (24 h) were only rarely reported (Figure 4E).

For pregabalin, the most common starting doses were 25, 50 and 75 mg/day (26.5%, 25.3% and 23.5%, respectively) (Figure 4F). Higher initial doses were less frequently selected, with 100 and 150 mg/day used in 8.2% and 15.3% of cases, respectively. Overall, the distribution of initial pregabalin doses differed significantly from a uniform pattern (*χ* ^2^(5) = 89.2, *p* < 0.001), with a moderate‐to‐large effect size (Cramér's *V* = 0.46). The maximum doses most frequently prescribed were 300 and 600 mg/day (38.5% and 32.7%, respectively) (Figure 4G). In contrast, only a small proportion of clinicians escalated treatment up to 900 mg/day (3.8%). Overall, the distribution of maximum pregabalin doses differed significantly from a uniform pattern (*χ* ^2^(5) = 105.5, *p* < 0.001), with a moderate effect size (Cramér's *V* = 0.37). Pregabalin titration was predominantly carried out on a weekly basis (*p* < 0.001; Cramér's *V* = 0.78), similar to gabapentin, with slower titration schedules used only occasionally (Figure 4H). The use of prolonged‐release (retard) pregabalin formulations was evenly distributed among clinicians, with 53.5% reporting their use and 46.5% reporting non‐use (*p* = 0.523) (Figure 4I). Twice‐daily administration (12 h) was the prevailing regimen, while once‐daily (24 h) and three‐times‐daily (8 h) schedules were much less frequent (Figure 4J).

### 3.6 Antidepressant Dosing Patterns

**FIGURE 5:**
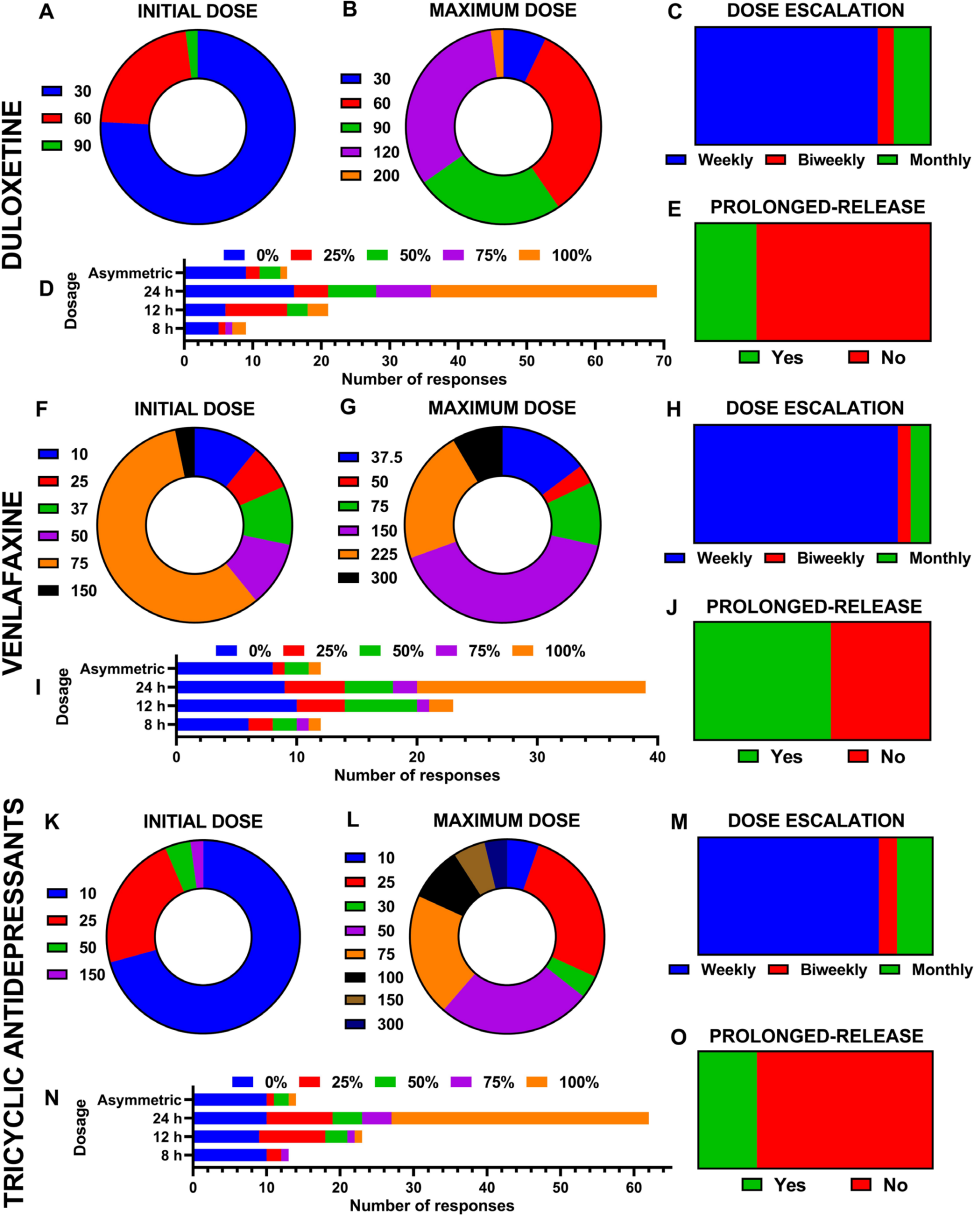
Dosage patterns of antidepressants. (A–E) Duloxetine: Initial and maximum doses (mg/day), titration schedules, use of prolonged‐release formulations, and preferred dosing frequency. (F–J) Venlafaxine: Initial and maximum doses, titration schedules, use of prolonged‐release formulations, and preferred dosing frequency. (K–O) Tricyclic antidepressants (amitriptyline): Initial and maximum doses, titration schedules, use of prolonged‐release formulations, and preferred dosing frequency.

For duloxetine, the most frequently reported initial dose was 30 mg/day (75.8%), followed by 60 mg/day (22.1%); higher doses (≥ 90 mg) were seldom prescribed (Figure 5A). This distribution differed significantly from a uniform pattern (*χ* ^2^(2) = 136.3, *p* < 0.001; Cramér's *V* = 0.68) (Figure 5A). The most common maximum doses prescribed were 60, 90 and 120 mg/day (33.3%, 24.8% and 32.6%, respectively), although a residual part of respondents (2.1%) escalated up to 200 mg/day (Figure 5B). This pattern was statistically significant (*χ* ^2^(4) = 56.1, *p* < 0.001; Cramér's *V* = 0.36). Dose titration was usually performed on a weekly basis (*p* < 0.001; Cramér's *V* = 0.65), with biweekly or monthly adjustments used rarely (Figure 5C). Extended‐release formulations of duloxetine were not used in clinical practice as they are not available (Figure 5D; *p* < 0.001). The preferred administration schedule was once daily (24 h), while twice daily (12 h) or three times per day (8 h) regimens were infrequent (Figure 5E).

Venlafaxine was typically initiated at 75 mg/day (57.6%) (Figure 5F). Other doses including 10, 25, 37.5 and 150 mg/day (10.9%, 7.6%, 9.8% and 10.9%, respectively) were less frequently used when starting the treatment (*χ* ^2^(5) = 96.0, *p* < 0.001; Cramér's *V* = 0.43). The maximum dose most frequently prescribed ranged from 150 to 225 mg/day (41.1% and 22.1%, respectively; *χ* ^2^(5) = 41.6, *p* < 0.001; Cramér's *V* = 0.29), with a minority (8.4%) escalating to 300 mg/day (Figure 5G). Weekly titration was the predominant approach (*p* < 0.001; Cramér's *V* = 0.76), while slower adjustments were scarce (Figure 5H). Prolonged‐release venlafaxine formulations were the most commonly prescribed (57.9%; *p* = 0.29), while immediate‐release formulations were used slightly less frequently. Venlafaxine was almost exclusively administered once daily, with other dosing frequencies being exceptional (Figure 5J).

For TCAs (amitriptyline), most of the specialists initiated therapy at 10 mg/day (70.7%), with fewer professionals starting at 25 mg/day (22.9%) or higher (50 mg/day; 4.3%) (Figure 5K). This pattern was statistically significant (*χ* ^2^(3) = 155.4, *p* < 0.001; Cramér's *V* = 0.61). Maximum prescribed doses typically ranged between 25, 50 and 75 mg/day (26.5%, 25.8% and 20.5%, respectively), although some reported titrating up to 100, 150 or 300 mg/day (9.1%, 5.3% and 3.8%, respectively) (Figure 5L). Again, this pattern was statistically significant (*χ* ^2^(7) = 54.9, *p* < 0.001; Cramér's *V* = 0.29). Weekly escalation was the standard practice (*p* < 0.001; Cramér's *V* = 0.64), with biweekly or monthly adjustments rarely employed (Figure 5M). Prolonged‐release formulations were not prescribed as they are not available (Figure 5N; *p* < 0.001). Administration was most often once daily (24 h), typically at night, with twice‐daily dosing (12 h) or three times per day (8 h) regimens rarely used (Figure 5O).

### 3.7 Influence of Clinical Experience and Expertise on Prescription Patterns

To explore whether clinical experience or expertise influenced prescribing behaviour, inferential analyses were conducted assessing the association between years of prescribing experience and the frequency of NP management (expertise) with key clinical decisions (Table 2). These included adherence to clinical guidelines, selection of first‐line agents, and preference for tramadol as a second‐ or third‐line treatment.

**TABLE 2:** Association between clinical experience, expertise and prescribing behaviour.

Predictor	Outcome	*χ* ^2^	df	*p* (asymptotic)	*p* (Monte Carlo)	Cramér's *V*
Years of prescribing experience	Adherence to guidelines (yes/no)	6.91	6	0.329	0.309	0.13
Years of prescribing experience	First‐line agent (top‐ranked)	11.94	12	0.451	0.442	0.14
Years of prescribing experience	Tramadol as most frequent 2nd/3rd‐line choice (yes/no)	1.70	3	0.636	—	0.09
Frequency of NP management	Adherence to guidelines (yes/no)	3.07	6	0.800	0.778	0.08
**Frequency of NP management**	**First‐line agent (top‐ranked)**	**25.41**	**12**	**0.013**	**0.023**	**0.20**
Frequency of NP management	Tramadol as most frequent 2nd/3rd‐line choice (Yes/No)	1.36	3	0.714	0.727	0.08

*Note:* Bold indicates statistically significant association. Chi‐squared tests were used to assess associations between categorical variables. Monte Carlo *p*‐values (5000 permutations) are reported when expected cell counts were < 5 in ≥ 20% of cells. Effect sizes were estimated using Cramér's *V*.

Abbreviations: df, degrees of freedom; NP, neuropathic pain.

Years of prescribing experience was not significantly associated with adherence to clinical guidelines (*χ* ^2^(6) = 6.91, *p* = 0.33), choice of first‐line agent (*χ* ^2^(12) = 11.94, *p* = 0.45), or preference for tramadol as the most frequently used second‐ or third‐line treatment (*χ* ^2^(3) = 1.70, *p* = 0.64). In all cases, effect sizes were small (Cramér's *V* ranging from 0.09 to 0.14), indicating a limited influence of prescribing seniority on treatment selection.

Similarly, the frequency with which clinicians managed patients with NP was not associated with adherence to clinical guidelines (*χ* ^2^(6) = 3.07, *p* = 0.80) or with preference for tramadol as a second‐ or third‐line option (*χ* ^2^(3) = 1.36, *p* = 0.71), again with very small effect sizes (Cramér's *V* < 0.10).

In contrast, a significant association was observed between the frequency of NP management and the choice of first‐line treatment (*χ* ^2^(12) = 25.41, *p* = 0.013). This association remained statistically significant after Monte Carlo correction (*p* = 0.023) and was associated with a small‐to‐moderate effect size (Cramér's *V* = 0.20), suggesting that clinicians with greater exposure to NP may adopt distinct first‐line prescribing strategies compared with those managing such patients less frequently.

## 4 Discussion

This nationwide survey provides an updated overview of analgesic prescribing patterns, decision‐making criteria, and dosage practices for NP among Spanish pain specialists. Consistent with the study objectives, our findings reveal moderate satisfaction with available pharmacological options, a strong reliance on clinical experience, partial adherence to international guidelines, and a heterogeneous approach to drug selection and treatment adjustment. Importantly, this study highlights that tolerance, which typically develops after 3–12 months of treatment, has been poorly documented. These findings have direct clinical implications, as they highlight specific gaps between guideline recommendations and real‐world prescribing patterns, thereby identifying clear opportunities to optimise pharmacological decision‐making, improve dosing strategies, and promote more evidence‐aligned management of neuropathic pain in specialised care.

The modest satisfaction reported by most pain specialists is consistent with the limited efficacy of pharmacological therapies documented in randomised controlled trials and meta‐analyses, where fewer than 40% of patients achieve clinically meaningful pain relief with monotherapy (Finnerup et al. 2015; Moore et al. 2009, 2014; Soliman et al. 2025). Our data confirm that this gap between expected and real‐world treatment effectiveness remains a central concern for specialists in Spain.

Prescribing decisions were predominantly guided by clinical experience, followed closely by adherence to guidelines. This finding is consistent with surveys conducted in other European countries: for example, in France, many general practitioners are aware of NP guidelines, but adherence remains low in practice, likely due to prioritisation of personal experience (Martinez et al. 2014; Rault et al. 2025). In Belgium, conventional analgesics such as paracetamol and NSAIDs continue to be the most frequently prescribed for NP, compared to guideline‐recommended treatments (antiepileptics, antidepressants, weak opioids) that are under‐utilised (Hans et al. 2007). Such reliance on personal experience may reflect the perceived limitations of standardised algorithms in complex comorbid populations commonly treated in pain units.

Guideline adherence was reported by slightly more than half of respondents, with NICE (NICE 2013), specialist evidence‐based guideline (Dworkin et al. 2007), and NeuPSIG (Finnerup et al. 2015; Soliman et al. 2025) being the most frequently cited. However, the relatively low overall uptake of these guidelines suggests a need for stronger dissemination strategies, national consensus recommendations or locally adapted protocols to bridge the gap between evidence and practice, as emphasised in a recent position paper calling for improved translation and implementation of best evidence in pain management (Pickering et al. 2025).

In terms of drug selection, gabapentinoids, followed by TCAs and duloxetine were the most frequently chosen first‐line options, in line with international guidelines (Attal et al. 2010; Finnerup et al. 2015; NICE 2020; Soliman et al. 2025). However, the continued high use of TCAs indicates that traditional prescribing habits remain influential, despite their less favourable safety and tolerability profile (Attal et al. 2010; Gustavsson et al. 2013; Attal and Bouhassira 2015; Moore et al. 2015). Tramadol emerged as the most frequently selected second‐line therapy, a finding that mirrors European practice and is consistent with EFNS (Attal et al. 2010), NeuPSIG (Soliman et al. 2025) and LONTS recommendations (Sommer et al. 2020), but that contrasts with recent NICE guidance which discourages routine opioid (including tramadol) use in NP (NICE 2020). This discrepancy with recent NICE guidance may reflect differences between European and Anglo‐Saxon approaches to so‐called ‘weak opioids’, with tramadol historically perceived in many European settings as a transitional or comparatively safer option. In addition, limited access to multidisciplinary and non‐pharmacological pain management strategies, as well as concerns regarding tolerability or contraindications of first‐line agents, may further contribute to reliance on tramadol in routine practice. While these factors cannot be directly assessed in the present survey, their consideration is important for interpreting real‐world prescribing patterns and informing future policy and guideline implementation efforts. The preference for pregabalin over gabapentin as the antiepileptic of choice is consistent with prescribing trends observed in France (Soeiro et al. 2025). Moreover, recent findings in the United Kingdom show that pregabalin prescribing continues to rise despite regulatory restrictions, while gabapentin prescription has levelled off (Connelly 2024). In contrast, other data show that gabapentin has historically predominated and that pregabalin prescription was later and less marked (Ashworth et al. 2023). In Denmark prescribing rates for both analgesics are broadly similar (Pottegård et al. 2025). These patterns highlight how regional differences in availability, cost, and prescriber perceptions have influenced gabapentinoid selection.

To our knowledge, previous clinician surveys have not quantified the phenomenon of tolerance in NP pharmacotherapy. Current guidelines emphasise periodic reassessment of pharmacological effectiveness due to the potential waning benefit over time (Finnerup et al. 2015; NICE 2020). Furthermore, opioid tolerance is well‐documented (Sommer et al. 2020). Our findings therefore add novel, practice‐based estimates of perceived tolerance that may develop over 3–12 months following analgesic prescription. Although underexplored in clinical trials, this tolerance may contribute to treatment dissatisfaction and high rates of therapy modification. The most frequent management strategy was dose escalation, which raises concerns regarding tolerability and potential safety risks. This highlights an urgent need for systematic evaluation of tolerance, as its recognition and characterisation could substantially improve clinical practice and inform future guideline recommendations.

Dosing patterns revealed substantial variability, particularly in the titration and ceiling doses of gabapentinoids and antidepressants. While most responding pain specialists followed guideline‐based initiation strategies, a considerable proportion prescribed outside the ranges typically evaluated in clinical trials. Similar heterogeneity has been reported in literature. For instance, studies describe common use of low initial doses in clinical practice (Kamble et al. 2017), while flexible‐dose trials suggest that higher pregabalin doses can improve outcomes (Serpell et al. 2017). Furthermore, meta‐analysis data indicate that intermediate gabapentin treatment programmes may achieve the best balance of efficacy and tolerability (Tsai et al. 2023). Narrative reviews likewise emphasise the broad therapeutic ranges used for antidepressants such as amitriptyline, duloxetine, and venlafaxine (Catalisano et al. 2024). Importantly, dose titration has been linked to better adherence and persistence in pregabalin‐treated patients, highlighting the value of physician awareness of dose recommendations and patient education on titration (Yeh et al. 2021). Collectively, these findings underscore the need for analgesic harmonisation and education about optimal dosing strategies.

In this context, our data suggest a systematic tendency to prescribe gabapentin at doses well below those commonly associated with optimal analgesic efficacy in neuropathic pain. This observation is consistent with previous real‐world studies reporting mean or median daily gabapentin doses of approximately 800–900 mg, substantially lower than the therapeutic ranges evaluated in randomised clinical trials (Gore et al. 2007; Johnson et al. 2013; Raouf et al. 2017). Such prescribing patterns support the concept of a potential ‘pseudo‐resistance’, whereby apparent treatment failure may arise not from intrinsic lack of drug efficacy, but from sub‐therapeutic dosing in routine clinical practice. Capsule burden, tolerability concerns, and adherence issues are likely contributors to this phenomenon, particularly in chronic treatment settings where dose escalation is required. In this regard, the limited availability of prolonged‐release gabapentin formulations in Spain may represent an additional structural barrier to dose optimisation, with potential implications for both treatment effectiveness and persistence. From a health‐policy perspective, improved access to prolonged‐release gabapentin formulations could facilitate dose optimisation, reduce pill burden, and potentially mitigate pseudo‐resistance in neuropathic pain management. The incorporation of inferential analyses adds complementary value by identifying associations between clinicians' experience, neuropathic pain caseload, and pharmacological treatment choices, thereby enhancing integration between the descriptive findings and the broader interpretative framework.

Beyond its national scope, this survey has relevant implications for the international pain community. The patterns observed in Spain are unlikely to represent an isolated situation and may reflect challenges shared by other European and international healthcare systems. In this context, our findings may serve as a benchmark for comparing prescribing practices and guideline adherence across countries. Identifying similar gaps elsewhere would support the existence of common barriers to evidence‐based implementation and highlight the need for coordinated educational and policy strategies to improve NP management.

### 4.1 Strengths and Limitations

This study has several strengths, including the participation of a well‐defined national sample of pain specialists, comprehensive coverage of prescribing behaviours, and detailed exploration of real‐world practices that are not usually captured in randomised trials. However, important limitations must be considered. The survey was conducted during the annual congress of the Spanish Pain Society but also disseminated through the Society's official communication channels. It is likely that most of the target audience were members of the Society, but this cannot be confirmed. The voluntary nature of participation introduces potential selection bias, which could lead to overrepresentation of clinicians with a stronger interest in NP or greater familiarity with guidelines, which could inflate estimates of adherence. Although changing the IP address could theoretically enable repeated entries of the survey, we consider this risk minimal considering the length of the survey, its voluntary nature, and the absence of incentives to intentionally provide duplicate responses. Reliance on self‐reported data carries risks of recall and social desirability bias, which could lead to underreporting of practices that deviate from recommendations. Moreover, the absence of patient‐level outcomes precludes evaluation of the actual clinical effectiveness of reported prescribing strategies, which limit interpretation of their impact. Furthermore, because the survey did not capture patient‐level clinical characteristics, interpretation of prescribing patterns cannot be stratified by neuropathic pain subtype or clinical complexity, thus limiting the granularity of our conclusions. Also, the survey focused exclusively on therapeutic approaches and did not consider whether the diagnosis of neuropathic pain was definitive. Noteworthy, the scarcity of neurologists is a defining characteristic of the Society's membership (and likely of many Pain Units) (Polo‐Santos et al. 2021). Finally, the exclusive focus on pain specialists increases the relevance of the findings for specialised units but reduces their generalisability to primary care or broader healthcare contexts. Also, given the exploratory nature of this survey, multiple statistical tests were performed without formal adjustment for multiple comparisons; therefore, the reported *p*‐values should be interpreted cautiously and primarily as hypothesis‐generating rather than confirmatory.

## 5 Conclusion

This survey highlights the variability and partial alignment of NP management in Spain with international recommendations. While gabapentinoids, followed by TCAs and duloxetine, remain central to therapy, reliance on personal experience, frequent use of tramadol, and the widespread recognition of tolerance illustrate the challenges of translating evidence into practice. Our findings support the need for national consensus guidelines, educational initiatives, and further research into poorly understood clinical phenomena such as tolerance, in order to harmonise care and improve outcomes for patients with NP.

## Author Contributions

The survey was designed collaboratively by all authors (members of the Neuropathic Pain Working Group of the Spanish Society of Pain), who contributed to the development and refinement of the questionnaire items. Data analysis and manuscript preparation were primarily carried out by M.Á.H., M.M.‐M., M.M.G., and M.C.R.‐C., A.S.‐A. was responsible for project coordination and funding acquisition. J.T., as a native English speaker, revised the manuscript for language and style. M.K., as an experienced statistician, performed and reviewed the statistical analyses. All authors critically reviewed the results, contributed to the discussion and approved the final version of the manuscript. All authors agree to be accountable for all aspects of the work.

## Funding

Gebro Pharma provided financial support for the survey. The sponsor had no role in the design, conduct, data analysis, interpretation or writing of this work.

## Conflicts of Interest

The authors declare no conflicts of interest. Although Gebro Pharma funded the survey, the sponsor was not involved in any aspect of the study or manuscript preparation.

## Supporting information


**Data S1:** ejp70246‐sup‐0001‐Supinfo01.pdf.

**Data S2:** ejp70246‐sup‐0002‐Supinfo02.pdf.
