# Supplementary material for: Prescription Trends and Clinical Decision‐Making in Neuropathic Pain Pharmacological Treatment: Results From a Cross‐Sectional Survey by the Spanish Pain Society
Source: Eur J Pain. 2026 Mar 10;30(3):e70246. doi: 10.1002/ejp.70246 (PMC12976174; doi:10.1002/ejp.70246)
Supplement: Supplementary file 1 — Data S1: ejp70246‐sup‐0001‐Supinfo01.pdf. [file EJP-30-0-s002.pdf]

SED
Sociedad Española del Dolor

Hábitos de prescripción para dolor neuropático dentro de la práctica clínica

Sección 1: Hábitos generales de prescripción y manejo del dolor neuropático

¿Qué tan satisfecho está con los resultados de los tratamientos farmacológicos actuales para el dolor neuropático en sus pacientes?

- ○ Muy satisfecho
- ○ Satisfecho
- ○ Neutral
- ○ Insatisfecho
- ○ Muy insatisfecho

¿En qué se basa para pautar los fármacos en dolor neuropático? (Elección múltiple)

- ☐ Publicaciones (datos clínicos)
- ☐ Recomendaciones de sociedades científicas (guías clínicas)
- ☐ Datos aportados por el comercial
- ☐ Datos preclínicos
- ☐ Experiencia propia

¿Qué factores considera al elegir un tratamiento farmacológico para el dolor neuropático? (Ordenar por prioridad)

Eficacia del medicamento

- Perfil de efectos secundarios
- Comorbilidades del paciente
- Preferencias del paciente
- Coste del medicamento

¿Sigue alguna guía clínica para tratar el dolor neuropático?

- ○ No
- ○ Sí

¿Con qué frecuencia revisa la mejoría de los pacientes tras iniciar el tratamiento para el dolor neuropático?

- ○ Entre 1 y 2 semanas
- ○ Entre 2 semanas y 1 mes
- ○ Entre 3 y 6 meses
- ○ Más de 6 meses
- ○ Según sea necesario

¿Cómo determina si un tratamiento farmacológico debe ser ajustado o cambiado? (Elección múltiple)

- ☐ Evaluación de la reducción del dolor
- ☐ Presencia de efectos secundarios
- ☐ Satisfacción del paciente con el tratamiento
- ☐ Mejoría en la funcionalidad y calidad de vida
- ☐ Coste-eficacia

¿Qué indicadores considera para decidir la continuación o interrupción de un tratamiento específico? (Elección múltiple)

- ☐ Eficacia sostenida del tratamiento
- ☐ Tolerancia del paciente al medicamento
- ☐ Ausencia de efectos adversos significativos
- ☐ Preferencias del paciente
- ☐ Comorbilidades y estado general de salud del paciente

¿Ha observado que sus pacientes tienen taquifilaxia al tratamiento (disminución de la eficacia tras la administración de unas pocas dosis)?

- ○ No
- ○ Sí

¿Cuándo aparece la taquifilaxia? (Indicar un número)

meses

¿Qué hace si hay taquifilaxia? (Ordenar por prioridad, arrastrando las opciones con el ratón)

- Subo dosis.
- Cambio a otro fármaco del mismo grupo.
- Cambio por otro fármaco de otro grupo del mismo nivel/escalón terapéutico.
- Inicio terapia combinada.
- Remito a otro compañero.

¿Hasta cuándo mantiene el tratamiento? ¿Cuándo se retira?

- ○ Cuando no mejora > 25%.
- ○ Cuando no mejora > 50%.
- ○ Cuando no mejora > 75%.

¿A partir de qué porcentaje de mejoría considera no necesario subir el tratamiento farmacológico?

- ○ Mejoría del 30%.
- ○ Mejoría del 50%.
- ○ Mejoría del 75%.

¿Con qué frecuencia cambia o ajusta el tratamiento cuando no se observa mejoría en el dolor neuropático?

- ○ Inmediatamente.
- ○ Después de 2-4 semanas.
- ○ Después de 1-3 meses.

◯ Según la respuesta del paciente.

Una vez llega al objetivo terapéutico

◯ Mantengo la misma pauta.

◯ Planteo modificar la pauta para facilitar la adherencia intentando reducir el número de cápsulas y/o ingestas mediante liberación prolongada.

¿Cuánto tiempo considera que hay que mantener el tratamiento una vez has llegado al objetivo terapéutico?

meses

Sección 2: Hábitos específicos en fármacos de primera línea

¿De los tratamientos recomendados como primer escalón, cuáles prescribe con mayor frecuencia por primera vez a un paciente con dolor neuropático? (Ordenar por prioridad, arrastrando las opciones con el ratón)

- Gabapentina.
- Pregabalina.
- Duloxetina.
- Venlafaxina.
- Antidepresivo Tricíclico (Ej.: Amitriptilina, Imipramina).

¿De los tratamientos recomendados como SEGUNDO ó TERCER escalón, cuáles prescribe con mayor frecuencia por primera vez a un paciente con dolor neuropático? (Ordenar por prioridad, arrastrando las opciones con el ratón)

- Tramadol.
- Parche de Capsaicina 8%.
- Parche de Lidocaína 5%.
- Lamotrigina.

Baclofeno.

Cannabinoides.

Crema de Capsaicina.

Oxcarbazepina.

¿Cuál es su antiepiléptico de prescripción preferido para el dolor neuropático?

- ◯ Gabapentina.
- ◯ Pregabalina.
- ◯ Lamotrigina.

¿Cuál es su antidepresivo de prescripción preferido para el dolor neuropático?

- ◯ Duloxetina.
- ◯ Venlafaxina.
- ◯ Antidepresivo Tricíclico (Ej.: Amitriptilina, Imipramina).

¿Qué hace si tiene efectos secundarios y eficacia parcial? (Ordenar por prioridad, arrastrando las opciones con el ratón)

Cambio por otro del mismo grupo. (switch) del primer escalón.

Coadministro otro de otro grupo de primer escalón (terapia combinada).

Coadministro un fármaco de segundo o tercer escalón.

Cambio por otro fármaco de segundo o tercer escalón.

Coadministro fármaco fuera de ficha técnica.

Cambio por otro fármaco fuera de ficha técnica.

Empleo técnica intervencionista.

¿Qué pasa si no tiene la eficacia analgésica deseada ni efectos secundarios? (Ordenar por prioridad, arrastrando las opciones con el ratón)

Aumento dosis.

Cambio por otro del primer escalón.

Coadministro otro del primer escalón.

Coadministro un fármaco de segundo o tercer escalón.

Cambio por otro fármaco de segundo o tercer escalón.

Coadministro fármaco fuera de ficha técnica.

Cambio por otro fármaco fuera de ficha técnica.

Empleo técnica intervencionista.

¿Prescribes directamente fármacos de 2º escalón sin pasar por el primero?

- ○ Sí.
- ○ No.

Sección 3: Hábitos específicos en fármacos de primera línea

Fármaco	Dosis Inicial Diaria (mg/día)	Dosis Máxima Diaria (mg/día)	Incrementos	Frecuencia (8h/12h/24h/Asimétrica)	Forma Retarda (Sí/No, ¿Por qué?)
Gabapentina	mg/día	mg/día	Incrementos semanales	% (8h)<br>% (12h)<br>% (24h)<br>% (Asimétrica)	○ Sí<br>○ No
Pregabalina	mg/día	mg/día	Incrementos semanales	% (8h)<br>% (12h)<br>% (24h)<br>% (Asimétrica)	○ Sí<br>○ No

Fármaco	Dosis Inicial Diaria (mg/día)	Dosis Máxima Diaria (mg/día)	Incrementos	Frecuencia (8h/12h/24h/Asimétrica)	Forma Retard[illegible] (Sí/No, ¿Por qué?)
Duloxetina	mg/día	mg/día	Incrementos semanales	% (8h)<br>% (12h)<br>% (24h)<br>% (Asimétrica)	Sí<br>N[illegible]
Venlafaxina	mg/día	mg/día	Incrementos semanales	% (8h)<br>% (12h)<br>% (24h)<br>% (Asimétrica)	Sí<br>N[illegible]
Antidepresivos Tricíclicos	mg/día	mg/día	Incrementos semanales	% (8h)<br>% (12h)<br>% (24h)<br>% (Asimétrica)	Sí<br>N[illegible]

Sección 4: Preguntas sociodemográficas

¿Con qué frecuencia trata a pacientes con dolor neuropático?

- Diariamente
- Semanalmente
- Mensualmente
- Raramente

¿Cuál es tu PRINCIPAL ámbito de trabajo?

- Público
- Privado

¿En qué Ciudad o Comunidad Autónoma trabajas?

Selecciona una opción

¿Cuál es tu edad?

- x ≤ 30 años
- 30 < x ≤ 40

- 40 < x ≤ 50
- 50 < x ≤ 60
- 60 < x ≤ 70
- x > 70 años

¿Cuántos años llevas prescribiendo?

- x ≤ 5 años
- 5 < x ≤ 10
- 10 < x ≤ 20
- > 20 años

¿Cuál es tu género?

- Masculino
- Femenino
- Otro
- Prefiero no decir

Finalizar encuesta
