# Supplementary material for: Prescription Trends and Clinical Decision‐Making in Neuropathic Pain Pharmacological Treatment: Results From a Cross‐Sectional Survey by the Spanish Pain Society
Source: Eur J Pain. 2026 Mar 10;30(3):e70246. doi: 10.1002/ejp.70246 (PMC12976174; doi:10.1002/ejp.70246)
Supplement: Supplementary file 2 — Data S2: ejp70246‐sup‐0002‐Supinfo02.pdf. [file EJP-30-0-s001.pdf]

SED
Sociedad Española del Dolor

Hábitos de prescripción para dolor neuropático dentro de la práctica clínica

Survey: Prescription Habits and Management of Neuropathic Pain

Section 1: General habits in prescribing and managing neuropathic pain

How satisfied are you with the results of current pharmacological treatments for neuropathic pain in your patients?
- Very satisfied
- Satisfied
- Neutral
- Dissatisfied
- Very dissatisfied

On what do you base your prescribing of drugs for neuropathic pain? (Multiple choice)
- Publications (clinical data)
- Recommendations from scientific societies (clinical guidelines)
- Information provided by pharmaceutical representatives
- Preclinical data
- Own experience

Which factors do you consider when choosing a pharmacological treatment for neuropathic pain? (Rank by priority)
- Drug efficacy
- Side effect profile
- Patient comorbidities
- Patient preferences
- Drug cost

Do you follow any clinical guideline to treat neuropathic pain?
- No
- Yes

How often do you assess patient improvement after starting treatment for neuropathic pain?
- Between 1 and 2 weeks
- Between 2 weeks and 1 month

- Between 3 and 6 months
- More than 6 months
- As needed

How do you determine whether a pharmacological treatment should be adjusted or changed? (Multiple choice)
- Assessment of pain reduction
- Presence of side effects
- Patient satisfaction with treatment
- Improvement in functionality and quality of life
- Cost-effectiveness

Which indicators do you consider to decide on the continuation or discontinuation of a specific treatment? (Multiple choice)
- Sustained treatment efficacy
- Patient tolerance to the drug
- Absence of significant adverse effects
- Patient preferences
- Patient comorbidities and general health

Have you observed tachyphylaxis to treatment in your patients (loss of efficacy after only a few doses)?
- No
- Yes

When does tachyphylaxis appear? (Indicate a number)
- Months

What do you do if tachyphylaxis occurs? (Rank by priority)
- Increase dose
- Switch to another drug in the same group
- Switch to another drug from a different group at the same therapeutic step/level
- Start combination therapy
- Refer to another colleague

Until when do you maintain the treatment? When do you withdraw it?
- When improvement is ≤ 25%
- When improvement is ≤ 50%
- When improvement is ≤ 75%

From what percentage of improvement do you consider it unnecessary to increase the pharmacological treatment?
- 30% improvement
- 50% improvement
- 75% improvement

How often do you change or adjust the treatment when no improvement in neuropathic pain is observed?
- Immediately
- After 2–4 weeks
- After 1–3 months
- Depending on patient response

Once the therapeutic goal is achieved:
- I maintain the same regimen
- I consider modifying the regimen to improve adherence by reducing the number of capsules and/or intakes with extended-release formulations

How long do you consider treatment should be maintained once the therapeutic goal has been achieved?
- Months

Section 2: Specific habits regarding first-line drugs

Among the recommended first-line treatments, which do you most frequently prescribe first to a patient with neuropathic pain? (Rank by priority)
- Gabapentin
- Pregabalin
- Duloxetine
- Venlafaxine
- Tricyclic antidepressant (e.g., Amitriptyline, Imipramine)

Among the recommended second- or third-line treatments, which do you most frequently prescribe first to a patient with neuropathic pain? (Rank by priority)
- Tramadol
- Capsaicin 8% patch
- Lidocaine 5% patch
- Lamotrigine
- Baclofen
- Cannabinoids
- Capsaicin cream
- Oxcarbazepine

Which is your preferred antiepileptic drug for neuropathic pain?
- Gabapentin
- Pregabalin
- Lamotrigine

Which is your preferred antidepressant for neuropathic pain?
- Duloxetine
- Venlafaxine
- Tricyclic antidepressant (e.g., Amitriptyline, Imipramine)

What do you do if there are side effects and partial efficacy? (Rank by priority)
- Switch to another first-line drug (same group)
- Add another first-line drug from a different group (combination therapy)
- Add a second- or third-line drug
- Switch to a second- or third-line drug
- Add an off-label drug
- Switch to an off-label drug
- Use interventional technique

What do you do if there is no analgesic efficacy and no side effects? (Rank by priority)
- Increase dose
- Switch to another first-line drug
- Add another first-line drug
- Add a second- or third-line drug
- Switch to a second- or third-line drug
- Add an off-label drug
- Switch to an off-label drug
- Use interventional technique

Do you directly prescribe second-line drugs without going through first-line ones?
- Yes
- No

Section 3: Specific habits regarding first-line drugs (dosing)

Drug – Initial Daily Dose (mg/day) – Maximum Daily Dose (mg/day) – Titration – Frequency (8h/12h/24h/Asymmetric) – Extended-release formulation (Yes/No, Why?)

- Gabapentin

- Pregabalin

- Duloxetine

- Venlafaxine

- Tricyclic antidepressants

Section 4: Sociodemographic questions

How often do you treat patients with neuropathic pain?
- Daily
- Weekly
- Monthly
- Rarely

What is your MAIN work setting?
- Public
- Private

In which city or autonomous community do you work?
- Select an option

What is your age?
- ≤ 30 years
- 31–40 years
- 41–50 years
- 51–60 years
- 61–70 years
- > 70 years

How many years have you been prescribing?
- ≤ 5 years
- 6–10 years
- 11–20 years
- > 20 years

What is your gender?
- Male
- Female
- Other
- Prefer not to say

End of survey
